# Mitochondrial Dysfunction in Congenital Heart Disease

**DOI:** 10.3390/jcdd12020042

**Published:** 2025-01-25

**Authors:** Julie Pires Da Silva, Mariana Casa de Vito, Carissa Miyano, Carmen C. Sucharov

**Affiliations:** Division of Cardiology, Department of Medicine, University of Colorado Anschutz Medical Campus, Aurora, CO 80045, USA; julie.piresdasilva@cuanschutz.edu (J.P.D.S.); mariana.casadevito@cuanschutz.edu (M.C.d.V.); cam523@nau.edu (C.M.)

**Keywords:** mitochondria, gestational diabetes, congenital heart disease, heterotaxy

## Abstract

Mitochondria play a crucial role in multiple cellular processes such as energy metabolism, generation of reactive oxygen species, excitation–contraction coupling, cell survival and death. Dysfunction of mitochondria contributes to the development of cancer; neuromuscular, cardiovascular/congenital heart disease; and metabolic diseases, including diabetes. Mitochondrial dysfunction can result in excessive reactive oxygen species, a decrease in energy production, mitophagy and apoptosis. All these processes are known to be dysregulated in cardiovascular diseases. The focus of this review is to summarize our current knowledge of mitochondrial dysfunction, including mitophagy and apoptosis, in pediatric congenital heart disease due to maternal diabetes or due to structural cardiac defects, with a focus on single-ventricle congenital heart disease. We also discuss recent mitochondria-targeted therapies for cardiovascular diseases.

## 1. Cardiac Mitochondrial Biogenesis

The heart is a highly metabolic active organ. Its oxygen consumption is the highest of any organ in the body. The heart requires a continuous supply of adenosine triphosphate to power its contraction, which is generated in the mitochondria. During embryonic development, the heart is one of the first organs to differentiate and function. It is composed of cardiomyocytes, smooth muscle cells, endothelial cells and fibroblasts. Cardiomyocytes proliferate during prenatal stages, with limited proliferative capacity postnatally. This is associated with changes in gene expression to an adult expression profile; cardiomyocyte structure maturation; and changes in mitochondria energy substrate utilization from glycolysis, as the main source of energy, to fatty acid oxidation (FAO), which produces ATP more efficiently, better supporting the increasing contractile demands of the mature cardiomyocyte [1].

Mitochondrial biogenesis requires a transcriptional network that encompasses the nuclear and mitochondrial genome in response to physiological changes during development. Studies have identified that the peroxisome proliferator-activated receptor-gamma coactivators (PGC-1α and PGC-1β) bind to nuclear receptors that regulate the transcription of Peroxisome proliferator-activated receptor (PPARγ), a nuclear receptor that plays a role in glucose and fatty acid metabolism [2]. Together, these coactivators and nuclear receptors act as important transcriptional players in the biogenesis process, and they are known to regulate genes involved in mitochondrial FAO and other lipid metabolic pathways. In addition to PPARγ, PGC-1α interacts with nuclear receptor proteins, such as nuclear respiratory factor-1 (NRF-1) and estrogen-related receptor (ERR), increasing transcription of targeted genes. NRF-1 regulates the expression of complexes involved in the electron transport chain (ETC), and its interaction with PGC-1 activates transcription factors associated with genes that mediate mitochondrial DNA (mtDNA) replication and transcription. ERRα supports mitochondrial biogenesis by regulating genes encoding medium-chain acyl-CoA dehydrogenase (MCAD), an enzyme involved in the first steps of FAO; the citric acid cycle (CAC); ATP synthesis, ETC and oxidative phosphorylation (OXPHOS). The enhancement of these complexes supports mitochondrial biogenesis and maturation [1].

## 2. Fuel Consumption in Cardiac Homeostasis and in Disease

**Figure 1:**
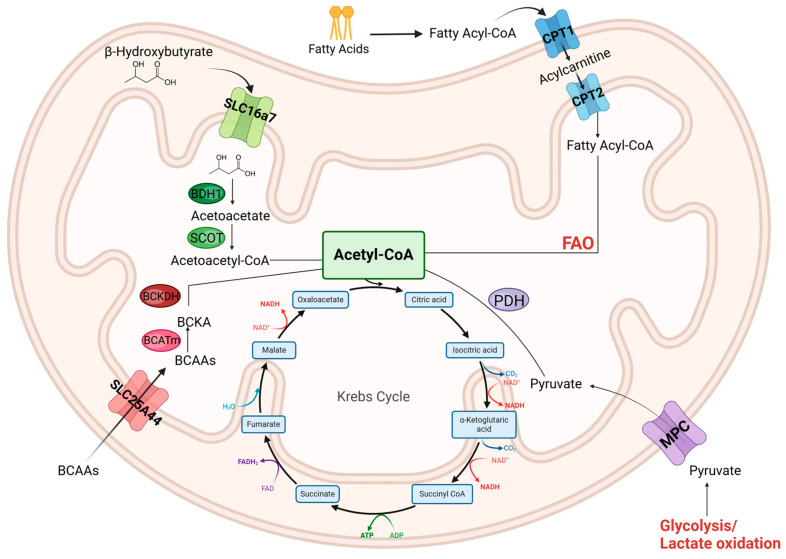
Substrate utilization of the myocardium. The heart utilizes various sources of fuel to drive adenosine triphosphate (ATP) production in the mitochondria. Fatty acid oxidation (FAO) is the main source of fuel. Once fatty acids are transported into the cytosol, carnitine palmitoyl transferase 1 (CPT1) adds a carnitine group on them, allowing for transport into the IMM. CPT2 then removes the carnitine group as it transports the fatty acids into the mitochondrial matrix (MM), where FAO takes place, yielding Acetyl-CoA. Glucose oxidation is still present in the mature cardiomyocytes, except at a lower rate. Glucose transporters introduce glucose and lactate into the cytosol, where they undergo glycolysis. The end product, pyruvate, gets transported into the MM by mitochondria pyruvate carrier (MPC), where pyruvate dehydrogenase (PDH) converts it into Acetyl-CoA. Ketones, such as 3-hydroxybutyrate, often play a small role in energy production in the mitochondria. As it is a small-carbon-chain molecule, it is easily defused though the OMM and then transported into the matrix by SLC16a7 transporter, where it undergoes ketolysis. B-hydroxybutyrate dehydrogenase 1 (BDH1) converts it into acetoacetate, followed by succinyl-CoA-oxoacid transferase (SCOT) conversion into acetoacetyl-CoA. This molecule gets cleaved into 2 Acetyl-CoA molecules. Branched-chain amino acids (BCAAs), under normal circumstances, also play a supporting role in mitochondrial metabolism. Different amino acids are transported into the matrix via BCAA symporter, where they are converted to ketoacids by branched-chain amino acid aminotransferase (BCATm) enzymatic activity. Branched-chain acid alpha-keto acid dehydrogenase (BCKDH) then converts these ketoacids into Acetyl-CoA. The Acetyl-CoA from all these fuel sources is responsible for feeding the Tricarboxylic acid cycle (Krebs cycle) that generates the reducing equivalents required to transfer electrons through the electron transport chain (ETC) complexes, resulting in adenosine triphosphate (ATP) production.

In mature cardiomyocytes, mitochondria mainly produce ATP by breaking down fatty acids through a process called fatty acid oxidation (FAO), which generates 40–60% of ATP. Other substrates, such as glucose-derived substrates, ketones and branched-chain amino acids (BCAAs), can also be utilized by the mitochondria but represent a secondary source of ATP in healthy cardiomyocytes, generating 20% to 40% (glucose oxidation), 10–15% (ketone oxidation) and 1–2% (BCAAs) of ATP [3]. In response to a pathological insult, there are metabolic adaptations to the changes in the cell environment that lead to cardiac mitochondria becoming dysfunctional, and in cardiomyocytes, becoming energy deficient. Although this process is not fully understood, unhealthy myocytes have a decreased ability to import fatty acids into the cells and into the mitochondria, reducing fatty acid availability, oxidation and ATP production. In addition, there is a decrease in enzymes that break down certain fatty acids, further declining FAO. As the pathology worsens, blood circulation is diminished, delivering less oxygen and nutrients to cardiomyocytes; therefore, under partial anaerobic conditions and nutrient depletion, the cells have to heavily rely on glucose oxidation for fuel, which leads to a lower ATP yield [4] (Figure 1).

In addition, recent systemic profiling of patients ranging from early- to end-stage heart failure (HF) have shown an increase in ketone body consumption [5], likely because the body and the heart become more energy starved. Ketones are produced endogenously, mainly by the liver, during periods of nutritional stress [6] and can be used as an alternative source of fuel in cardiomyocytes. Ketone bodies, such as β-hydroxybutyrate, have a similar turnover rate in producing ATP when compared to FAO and a higher rate than glucose oxidation [7]. However, since it is not readily available in large quantities in the body, it is only used in extreme nutrient deficiency conditions. Evidence is still being gathered to support the idea that ketone use is increased and beneficial in heart failure, and that treatments that drive the overproduction of ketone bodies can relieve HF symptoms (Figure 1).

Under cellular stress conditions such as extensive mitochondrial dysfunction, there are certain cellular process that take place in order to help establish homeostasis. Initial efforts include mitophagy, which is a process that allows the cell to rid itself of damaged mitochondria, and mitochondrial biogenesis, which repopulates myocytes with healthy mitochondria, improving energy production. Throughout the progression of heart disease, these processes may not be sufficient to improve the extent of the damage, and in this case, cellular death pathways will be activated to clear out these myocytes. Such processes are important to understand the metabolic environment of the heart.

## 3. Mitophagy in Cardiomyocytes

Mitophagy plays an important role in maintaining cell homeostasis by clearing out damaged mitochondria that are overproducing reactive oxygen species (ROS) and free radicals. Previous studies have shown that excessive ROS not only causes oxidative damage in the cells’ DNA, but is even more damaging to mitochondrial DNA, which is less protected and therefore more susceptible to stress, resulting in disease-causing mutations. Mitophagy in cardiac cells is especially important, as healthy mitochondria play a key role in the energy required to sustain the cardiac workload. Mitochondria compose 20% to 40% of the volume of adult cardiomyocytes, and a mechanism that ensures healthy mitochondria is key to proper cardiac function [1].

Mitophagy is a selective type of autophagy, where the mitochondria that are damaged or undergoing excessive stress are targeted for degradation. Mitophagy is also necessary during cardiac maturation, as there is a need to replace mitochondria that use carbohydrates as their energy source with ones that primarily use fatty acids as substrates [1]. The most common mitophagic pathway in several cell types is the PINK/Parkin-dependent mitophagy pathway [8]. In healthy cells with low cellular stress and well-functioning mitochondria, their membrane potential is polarized, allowing serine-threonine kinase PINK1 (PTEN-induced putative kinase 1) to travel into the mitochondrial intermembrane space though the TIM/TOM protein complex (translocase of the inner/outer membrane), where it can be degraded. Maintaining low levels of PINK1 in the cytosol is essential to keep mitophagy mostly dormant, as the kinase is responsible for phosphorylating and activating Parkin, an E3 ubiquitin ligase, which initiates mitophagy. In diseased hearts, cellular stress often leads to depolarization of the mitochondrial membrane, resulting in the inactivation of the TIM/TOM complex. Thus, PINK1 cannot be degraded, recruiting Parkin in an unregulated manner, and resulting in polyubiquination of membrane proteins [9]. This ensures that damaged mitochondria are targeted by the autophagosome–lysosomal pathway that will degrade the organelle via a pH-mediated protein denaturing process. In cardiomyocytes, the role of Rab (Ras analogue in brain) proteins in the PINK/Parkin pathways has been defined as meaningful for mitochondrial clearance. Following substantial mitochondrial damage and accumulation of PINK and Parkin on the OMM, Rab guanine nucleotide exchange factor 1 (RabGEF1) is recruited to the mitochondria, signaling Rab5 to do the same. Rab5 then recruits Rab7 via MON1/CCZ1 (MC1) complex, assembling vesicles and autophagic membranes to complete mitophagy [10].

## 4. Mitochondria in Cardiac Cell Death

The process of programmed cell death is a regulated process important for maintaining cell and system homeostasis. In vital organs, such as the heart, cell death can be associated with pathological processes, including myocardial infarction and heart failure. In cardiac tissue, there are six types of cell death, which are regulated by extrinsic or intrinsic signals and pathways [11]. The more common forms of cell death include apoptosis and autophagic cell death; however, cardiomyocytes also undergo ferroptosis, pyroptosis, necrosis and mitochondrial-mediated necrosis. These are associated with organ inflammation, as intracellular content is often released into the entire system, which furthers the process of heart failure. Since the regeneration capacity of adult cardiomyocytes is limited, cardiomyocyte cell death is accompanied by proliferation of non-contractile cells, such as fibroblasts, which can result in fibrotic tissue formation [12].

Apoptosis, although important for proper cell homeostasis, also contributes to pathogenesis in cardiac diseases. Its activation can be through extrinsic ligands that bind membrane receptors of targeted cells, or intrinsically, most likely due to oxidative stress. In heart failure, pressure overload often leads to oxidative stress [13] that causes mitochondria to release cytochrome C into the cytosol, which initiates a cascade of events that lead to apoptotic cell death. Cytochrome C and Bcl-2 family proteins, specifically, pro-death proteins, which include BAX, BAK and BID [14], create a complex that activates APF-1 and Caspase-9. This complex then activates effector Caspase-3 that signals the final processes of apoptosis [15]. The activation of death receptors in the extrinsic pathways leads to conformational changes, which allow the adaptor protein Fas to activate Caspase-8. This process then activates Caspase-3, which follows the same final apoptotic stages as described in the intrinsic pathway [11]. An interesting study showed the detrimental effects of apoptosis blockade or over activation. In this study, inactivation of Caspase-8 resulted in cardiomyopathy, whereas over activation caused thickening of the heart wall due to edema [16]. In summary, regulated apoptosis is essential for heart health.

Cell death by necrosis is another important mechanism in cardiomyocytes; it is usually triggered by the TNF family of cytokines that bind to death receptors on the outer membrane of the cells. The activation of these receptors forms a complex of Fas-associated death proteins that either activates or deactivates Caspase-8; the former leads to apoptosis, and the later activates the necrotic pathway. Inactive Caspase-8 allows kinases to phosphorylate proteins, starting with receptor interacting kinases (RIP1 and 3) and culminating with mixed-lineage kinase domain-like (MLKL) [17]. RIP3 and MLKL form hetero-oligomers, and the later also forms homo-oligomers that are translocated to the cell membrane by heat shock proteins (HSPs). This acts as a signal for cell surface proteases, mainly from the A Disintegrin and Metalloprotease family (ADAM), to aggregate on the membrane. Activated ADAMs break down membrane proteins and form nonspecific pores that result in leakage of cell contents. This process causes systemic inflammation, which can promote cell death in neighboring cells [18]. In cardiomyocytes, mitochondria-mediated necrosis is usually the result of extensive oxidative stress or calcium buildup due to alterations in the mitochondrial permeability transition pores (MPTPs). MPTPs are calcium-regulated channels located across the inner and outer mitochondrial membrane that help maintain membrane potential in the organelle. Calcium overload and oxidative stress favor calcium-induced opening of MPTP and disrupt the membrane potential in the mitochondria, disrupting ATP Synthesis. The shift in membrane potential results in the flow of molecules into the inner mitochondrial membrane, causing its rupture and release of its contents, such as cytochrome C, into the cytosol [19]. This process triggers the same apoptotic or necrotic pathways discussed above.

Pyroptosis, which has been shown to play an important role in diabetic cardiomyopathy, atherosclerosis and dilated cardiomyopathy, is only induced in the presence of abundant mitochondrial ROS, and is activated by the presence of pathogenic-associated molecular patterns (PAMPs) or damage-associated molecular patterns (DAMPs). The cell responds to these stimuli by forming inflammasomes, most commonly composed of Nod-like receptor protein (NLRP3), adaptor protein ASC and pro-caspase 1, which activates caspase-1 [20]. This caspase cleaves Gasdermin D (GSDMD) proteins into two, and the N-terminal can bind to phosphoinositide lodged in the cell membrane. Similar to the final stages of necrosis, the oligomerization of these lipids forms pores in the membrane that results in leakage of cellular contents [11].

There are other less well understood cell death mechanisms, such as ferroptosis, that have been found to be activated in cardiomyocytes. Interestingly, cells that undergo ferroptosis have abnormal mitochondria that present increased membrane density and lower volume [21]. In healthy myocardial cells, Fe^3+^ bound to transferrin proteins is transported into the cells via transferrin receptor-1 (TFR-1), forming endosomes. Fe^3+^ is reduced to Fe^2+^ by a metalloreductase enzyme and is transported into the cytoplasm, as needed, by a divalent metal transporter-1 (DMT-1), present in these endosomes. In addition, ferritin light chain (FLT) and ferritin heavy-chain-1 (FHC-1) form a protein complex that oxidizes Fe^2+^ back into Fe^3+^ and binds to it, reducing the levels of free iron in the cytoplasm [22]. Often times, pathologies in the heart cause disturbances in blood circulation, such as reperfusion, which can result in an overload of iron in cardiac cells overwhelming these iron storage systems and resulting in iron excess. Iron is known to drive ROS production; therefore, the accumulation of iron in stressed cells could be detrimental. When iron gets pumped into the mitochondria, OXPHOS generates an overproduction of ROS and hydrogen peroxide (H_2_O_2_), resulting in the accumulation of lethal lipid hydroperoxides. Cardiomyocytes in diseased states have an inhibition of glutathione peroxidase-4 (GPX-4) enzymes, which would otherwise inhibit excess peroxides, preventing lipid peroxidation. The lack of GPX-4 in addition to increased ROS causes further oxidative stress and could trigger any of the cell death pathways mentioned [23].

## 5. Mitochondrial Dysfunction Associated with Left–Right Asymmetry

During normal cardiac development, a conserved ciliated left–right (L–R) organizer utilizes cilia, hair-like organelles, to generate and sense the directional flow of extraembryonic fluid, which eventually results in asymmetric heart looping. Due to the role of cilia in L–R patterning, cilia mutations can result in the pathogenesis of heterotaxy (HTX), which is characterized by abnormal L–R relationships and is considered a cause of heart malformations and congenital heart disease (CHD) [24]. The target-of-rapamycin (TOR) signaling pathway, which influences mitochondrial biogenesis, also modulates cilia size and function during zebrafish development [25]. Importantly, zebrafish with inhibited TORC1 signaling displayed HTX-like phenotypes, illustrating the importance of this pathway in L–R asymmetry.

Interestingly, mitochondrial biogenesis is one of the processes that mTOR regulates, and a recent study found that mtDNA content in human blood is lower in HTX patients compared to healthy controls. In contrast, patients with CHD without visceral situs anomalies exhibited mtDNA levels similar to healthy controls. These findings led to a study to investigate the role of mitochondrial function in zebrafish embryos, human fibroblasts, and *Tetrahymena thermophilia* [26]. In this study, developing zebrafish embryos were treated with the mitochondrial biogenesis stimulator, isoflavone 3-(2,4-dichlorophenyl-7-hydroxy-4H-chromen-4-one) (DCHC), or the mitochondrial respiration inhibitor, rotenone. The authors showed that mitochondrial homeostasis is essential for proper establishment of L–R asymmetry, in that increased biogenesis reduced cilia length, and respiration inhibition resulted in elongated cilia. Shortening or elongation of cilia disturbs fluid flow, negatively affecting symmetry, and potentially leading to CHD. Finally, an exome analysis from 285 HTX patients and examination of 1174 mitochondria-associated genes identified a significant increase in damaging variant burden in the HTX cohort compared with 1000 Genomes controls. Exome analysis revealed rare genetic variants in genes related to lipid metabolism and ROS regulation (*ACOX1*), DNA repair (*APEX2*), and mitochondrial cardiolipin transport (*TAZ*), amongst many others. Knockdown of candidate genes from this analysis resulted in ciliopathy-like phenotypes and cilia elongation in zebrafish. Altered mitochondrial function as a result of altered ciliogenesis appears to be an underlying cause of HTX and should be further studied to investigate if mitochondrial function is altered in these patients. Interestingly, studies showed that in 17.5% of cases of HTX, the overall organ situs is right-sided and therefore more likely associated with specific CHDs such as single-ventricle congenital heart disease (reviewed in [27]).

## 6. Mitochondrial Dysfunction in Congenital Heart Disease

There is a large and growing body of evidence for mitochondrial dysfunction and impaired cellular energy production in adult heart failure (HF), which is marked by a decrease in cellular adenosine triphosphate (ATP) and phosphocreatine, shifted substrate selectivity, increased reactive oxygen species (ROS) production and decreased expression in individual electron transport complexes [28]. In the CHD population, the molecular mechanisms underlying HF are not well understood, and treatments are often based on adult HF therapies [29]. Single-ventricle (SV) congenital heart disease is a CHD that comprises an array of congenital cardiac malformations defined by severe underdevelopment of one ventricle (for a comprehensive review see [30]). Children born with SV undergo surgical interventions that allow the single ventricle to be the only functional pump to actively or passively deliver blood to the systemic and pulmonary circulations, but failure of the morphological right ventricle (RV) is common, and HF is a leading cause of death and indication for transplant in this population. Pressure overload of the RV has been shown to result in increased oxidative stress, reduced angiogenic response and increased activation of apoptotic pathways [31]. Metabolic adaptations of the RV in response to pressure overload include an increased reliance on glycolysis, leading to an energy-starved state; abnormalities in mitochondrial complexes I, III and IV; and a progressive decline in mitochondrial DNA [32].

In a study published in 2023, our group showed mitochondrial dysfunction in the hearts of patients with heart failure due to SV heart disease (SVHF) [33]. In this study, we evaluated RV tissue from patients with SVHF and age-matched biventricular non-failing (BVNF) healthy controls. The majority of the hearts evaluated in that study were from patients transplanted after the Fontan palliation. Similar to what we observed in the pediatric and adult Dilated Cardiomyopathy (DCM) populations [34], SVHF hearts displayed impaired mitochondrial function associated with altered metabolism. Additionally, we quantified carnitine palmitoyl transferase (CPTI and CPTII) activity, which are required for FA transport into the mitochondria, and found a significant decrease in CPTI and CPII enzymatic activity in the SVHF population. However, FAO was not assed in this initial study. As highlighted in the accompanying editorial [32], this is a limitation of the study, as the major metabolic shift observed in these hearts is associated with fatty acid processing. Importantly, to our knowledge, CPT activity has not been investigated in human heart failure due to other causes.

**Figure 2:**
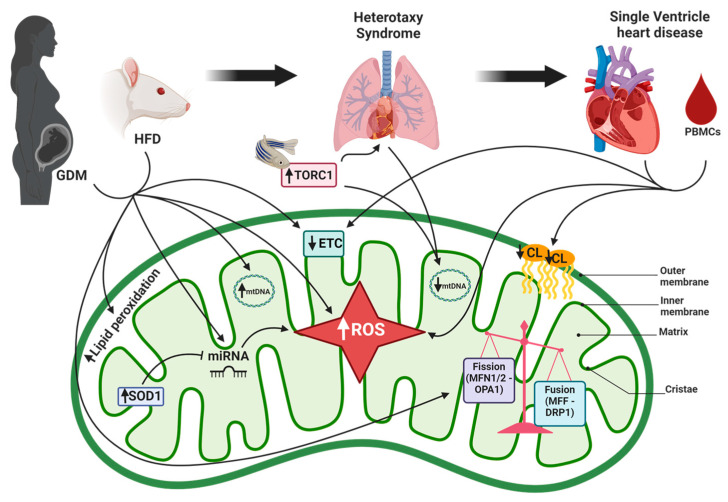
**Mitochondrial dysfunction associated with gestational diabetes, heterotaxy syndrome and single-ventricle disease.** Gestational diabetes mellitus (GDM) has been associated with heterotaxy syndrome and single-ventricle heart disease. GDM and the high-fat diet (HFD) rat model lead to a mitochondrial dysfunction characterized by an increase in lipid peroxidation, increase in mtDNA copy number and a pro-fission pattern by increasing expression of mitofusin 1/2 (MFN) and optic atrophy 1 (OPA1). The electron transport chain (ETC) is disrupted, and an increase in reactive oxygen species ROS occurred during GDM and single-ventricle heart disease. An overexpression of superoxide dismutase 1 (SOD1) in rat repressed GDM-regulated miRNAs and blunted the increase in ROS induced by GDM. Inhibition of rapamycin complex 1 (TORC1) in zebrafish induces a heterotaxy phenotype and modulates mitochondrial biogenesis by decreasing mtDNA content. In addition, blood from heterotaxy patients presents a lower mtDNA content. Myocardium and peripheral blood mononuclear cells (PBMCs) from single-ventricle patients show a decrease in mitochondrial respiratory function and cardiolipin (CL) content, which is associated with an increase in ROS. MFF, mitochondrial fission factor; DRP1, dynamin-related protein 1. This figure was created using Biorender.com.

Mitochondrial function is highly dependent on the protein and lipid composition of the mitochondrial membrane (reviewed in [35]). Of the lipids that compose the mitochondria membranes, cardiolipin (CL) is mostly found in the inner mitochondrial membrane. CL is a dimeric phospholipid specific to the inner mitochondrial membrane, whose function involves stabilizing the mitochondrial membrane, mitochondrial protein import and autophagy/mitophagy. Furthermore, CL is thought to stabilize the respiratory supercomplex of the mitochondria necessary for proper electron flow ATP generation [36]. Once cardiolipin is biosynthesized, its acyl chain is modified via CL remodeling enzymes, and the final product is known as a mature cardiolipin. Failure to properly remodel CL has been linked to mitochondrial dysfunction [37]. In addition, CL is known to be essential in the activation of the intrinsic apoptotic pathway by being selectively oxidized in response to oxidative stress [38]. The functional implications of its synthesis and remodeling have been debated, but it is now known that improper synthesis and remodeling, resulting in aberrant CL profiles, can result in cardiac disorders such as Barth Syndrome and cardiomyopathy, as well as mitochondrial dysfunction [39]. In a study by our group that examined CL profiles in SV heart samples, total CL and tetralinoleoyl cardiolipin, the most abundant form of mature CL in the heart, were lower in SV heart failure (SVHF) RV myocardium compared to age-matched biventricular non-failing control RV myocardium, even though the mitochondrial copy number was not significantly different between the groups (Figure 2). These results indicate alterations in CL, independent of mitochondria content in SV hearts, and suggest mitochondrial dysfunction is a contributing factor in the pathology of SVHF [37]. Similarly, we have shown that cardiolipin is altered in pediatric DCM [40].

A recent study showed that, in response to severe cardiac hypertrophy due to pressure overload in complex CHD (tetralogy of Fallot, pulmonary stenosis or double-chambered RV), lipid peroxidation was increased, which was associated with lower oxidative phosphorylation and structural damage of the mitochondria [41]. Furthermore, in a mouse model of RV hypertrophy and failure due to pressure overload, there was a decrease in electron transport chain activity, which was associated with alterations in mitochondrial protein content and changes in gene expression related to mitochondrial function [42]. Collectively, these studies indicate that mitochondrial function is a key component of CHD, and may be an important target of therapy for this population.

## 7. Mitochondrial Dysfunction Associated with CHD Due to Diabetes Mellitus

CHD can be the result of Diabetes Mellitus (DM) and maternal obesity. It has been postulated that transmission of maternal factors during pregnancy influences the risk of CHD in the offspring. These factors can include transmission of metabolites through the bloodstream or abnormal placental vascular supply due to obesity (Reviewed in [43]). Importantly, in CHD due to DM, mitochondrial dysfunction has been observed in association with increased oxidative stress [44]. Changes in gene expression in response to oxidative stress, especially in genes essential for normal cardiac development, have been demonstrated in response to maternal hyperglycemia [45]. To investigate the molecular mechanisms involved in the negative effects of human maternal diabetes on embryonic development, a recent study using a murine type 1 DM model identified 149 microRNAs that were significantly altered in the developing heart in a mouse model of pre-gestational DM (PGDM). The authors showed that overexpression of superoxide dismutase 1 (SOD1), a mitochondrial antioxidant enzyme, in the DM mouse blunted the changes in PGDM-regulated miRNAs and their target genes, highlighting the contribution of oxidative stress to changes in gene expression in the developing heart in response to PGDM [46] (Figure 2).

In a study that examined effects of diabetes and a high-fat diet (HFD), both separately and in combination, HFD-exposed Sprague-Dawley rats had an increased mitochondrial copy number, increased lipid peroxidation and mitochondrial dysfunction (Figure 2). Rats that developed diabetes had impaired glycolytic and respiratory capacity, in addition to a reduced proton leak. Rats exposed to HFD and diabetes during development were the most severely affected and had cardiomyocytes with fragmented and poorly charged mitochondria with few fusion or fission events [47]. A second study showed that developing cardiomyocytes exposed to HFD and/or diabetes had impaired fusion and fission events [48]. HFD during pregnancy caused an imbalance of fusion and fission in a sex-dependent manner in newborn offspring cardiomyocytes, where female mice had higher expression of fusion proteins, mitofusin 1 (MFN1), mitofusin 2 (MFN2) and optic atrophy 1 (OPA1) but not dynamin-related protein 1 (DRP1) or mitochondrial fission factor (MFF), possibly leading to a cardioprotective effect (Figure 2). Mitofusin function is dependent on post-translational modifications (PTMs), more specifically, ubiquination. The authors identified multiple MFN1 species including an active species at 70 kDa and an inactive (di-ubiquinated) species at 86 kDa. Analysis of both MFN species uncovered a male-specific diabetes- and diet-related decrease in expression of the active species in combination with an increase in the expression of the inactive species. These results are consistent with di-ubiquination and inactivation of the fusion protein, leading to the lower expression of MFN1 in male rats. Diabetic pregnancy impaired fusion (50%) more than fission (28%), which led to pro-fission imbalance as well as shorter and wider cardiac mitochondria. When HFD and diabetes were combined, cardiac mitochondrial dysfunction was exacerbated due to decreased respiratory capacity and increased oxidative stress. This study demonstrated the possibility of mitochondrial dynamism as a regulator in the development of CHD in infants born to diabetic or obese mothers.

## 8. Mitochondrial Dysfunction Associated with Cyanosis

Patients with CHD can sometimes experience a drop in circulating oxygen, and while it is established that CHD is accompanied by mitochondrial dysfunction, it is important to understand the impact of cyanosis on mitochondrial health. Roughly one-third of cardiomyocytes are occupied by mitochondria, and most of the ATP produced comes from aerobic oxidative phosphorylation. Naturally, limiting the amount of oxygen present in an environment where mitochondria are already dysfunctional can cause more damage. Unfortunately, the progression of dysfunction in this organelle often goes unnoticed until the hearts starts failing, as there are limited studies identifying early markers that could help diagnosis and improve patient outcomes. A study evaluating the effects of acyanotic and cyanotic CHD on mitochondrial dysfunction found that the cyanotic patients had lower mitochondrial density and ATP production. These same patients, post surgery, had higher levels of markers of heart disease such as troponin I, myoglobin and creatinine kinase-MB (CKMB), suggesting cyanotic patients have a higher chance of cardiac damage [49].

## 9. Mitochondrial-Targeted Therapies

**Figure 3:**
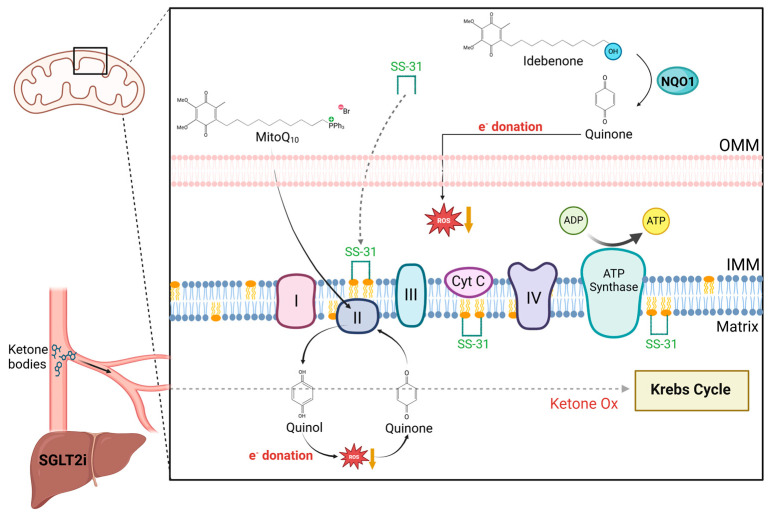
Mitochondrial targeted therapies for the failing heart. Sodium-glucose transport protein 2 inhibitor (SGLT2i) has been shown to increase endogenous production of ketone bodies in the liver. This likely results in an increase in circulating ketones bodies, which provides the heart with an additional source of fuel to support the impaired mitochondrial metabolism. Elamipretide (SS-31) is a peptide that can mediate the cardiolipin remodeling, known to play a role in the mitochondrial dysfunction present in pediatric heart failure. It can defuse into the outer mitochondrial membrane (OMM) and anchors the cardiolipin in the inner mitochondrial membrane (IMM) to stabilize the electron transport chain (ETC) complexes. Antioxidants such as MitoQ and Idebenone can interact with superoxides by donating electrons and detoxifying them. MitoQ is composed of quinone conjugated to a lipophilic triphenylphonium cation (TTP^+^) that allows it to travel into the mitochondrial matrix via membrane potential. There, it interacts with complex II, turning it into quinol molecules that can donate electrons to reactive oxygen species (ROS), reducing them. This donation results in quinone molecules that can cycle back through complex II to make more quinol. Idebenone gets directly turned into quinone’s active form in the cytoplasm by quinone oxireductase 1 (NQO1). Activated quinone molecules can donate electrons to ROS and free radicals, limiting their harmful interactions with other molecules.

With growing evidence of the role mitochondrial dysfunction has in cardiovascular diseases, there is an opportunity to explore mitochondrial targeted therapies as a potential treatment for patients with HF. Sodium-glucose Transport Protein 2 inhibitors (SGLT2is) have become a drug of interest in HF, as they were initially found to alleviate HF symptoms in patients with DM [50]. This compound, prescribed as a diuretic, acts by binding to the SGLT2 receptors found in the renal tubules. As a result, it reduces the absorption of glucose back into the bloodstream and promotes urinary glucose excretion [51]. Upon observing improvement of HF symptoms, studies investigated a potential cardiac effect. Although these studies are still ongoing, cardiac effects seem to be a consequence of its diuretic effects. In addition, studies have shown that this inhibitor can indirectly affect the heart by increasing the endogenous production of ketone bodies in the liver. The availability of circulating ketones may provide cardiac mitochondria with an extra source of fuel, which can increase ATP production and reduce ROS [52], improving mitochondrial stress in these dysfunctional cardiomyocytes (Figure 3).

Studies on Barth Syndrome suggest that mitochondria targeted therapies may be an optimum treatment to improve cardiac function. One of these compounds, Elamipretide, or SS-31, can improve mitochondrial function, and more significantly, complex I function. The peptide targets CL, which, as previously mentioned, is decreased and improperly remodeled in CHD due to the single-ventricle physiology [53], which can result in ETC supercomplex instability, which further decreases ATP and increases ROS production. Elamipretide can stabilize CL within the mitochondrial membrane acting as an anchor. This brings the ETC complexes closer together, allowing a more efficient electron flow, which in turn increases ATP yield (Figure 3).

Another important class of mitochondrial-targeting therapies are antioxidants. Mito-TEMPO is composed of peperdine nitroxide (TEMPO), a superoxide dismutase conjugated to a lipophilic triphenylphonium cation (TTP^+^) that allows this compound to enter the mitochondrial matrix driven by its membrane potential. There, Mito-TEMPO can reduce oxidative damage [54]. Mitochondria-targeted coenzyme Q10 (MitoQ) has also been established as an important antioxidant. MitoQ is composed of quinone and also requires TTP^+^ to enter the mitochondrial membrane [55], where it is reduced to quinol by complex II in the ETC and can also interact with superoxides, such as peroxidized lipids. This interaction oxidizes the molecules back to its quinone form; then, it is recycled through complex II back into its quinol form [55]. Lastly, recent studies have identified Idebenone, a coenzyme Q10 analogue, characterized by increased intestinal absorption and bioavailability, as well as smaller lipid sidechains with higher polarity. Therefore, it can be more effective than coenzyme Q10. Idebenone gets a two-electron reduction to an active hydroquinone form by quinone oxireductase 1 (NQO1), allowing it to detoxify free radicals [56] (Figure 3).

## 10. New Lines of Intervention

Mortality rates of infants with CHD are still high [57]. Surgical intervention for CHD may be classified into three categories: (i) palliative, (ii) reparative or (iii) corrective, with the aim to regain normal heart function and/or decrease adverse symptoms [58,59]. Although these surgeries improve the survival of CHD patients, heart failure is common, resulting in the need for heart transplantation [60]. Unfortunately, only 500 pediatric heart transplants are performed annually worldwide [61], highlighting the need to develop parallel strategies. A new and exciting potential therapy is called mitochondrial transplantation (reviewed in [62,63]). Ermani et al. were the first to develop this new therapy, which consists of replacing damaged native mitochondria with viable and competent mitochondria from non-ischemic autologous tissue directly in the injured tissue. In a model of rabbit, porcine and human-induced pluripotent stem cells (iPS)-derived cardiomyocytes, the authors showed an increase in energy production, decrease in cell death and upregulation of pathways for cellular respiration immediately after mitochondrial transplantation [62,63]. This technique has the possibility to improve mitochondrial structure and function that are altered in patients with CHD.

## 11. Gaps in Knowledge

In this review, we provided evidence that implicates mitochondrial biology (oxidative stress, mitochondrial dynamics, cardiolipin and energy sources) as possible mechanisms involved in the pathogenesis of GDM, HTX and SV congenital disease. These studies have improved our understanding of mitochondrial function in metabolic and SV congenital heart disease.

We have extensively discussed the association between GDM, HTX and SV congenital heart disease and their respective link to mitochondria. However, there are no studies showing that mitochondria are the common link in the development of these diseases. The body of literature on mitochondrial dysfunction related to these diseases implies a decrease in mitochondrial respiration and an increase in the production of ROS [26,31,45,46,64,65]. Further in vitro, animal and clinical studies must be conducted to investigate the causative role of mitochondria. The development of new therapeutic approaches that specifically target the mitochondria may improve cardiac function and/or delay transplantation in this vulnerable population.
